# Therapies from Fucoidan: An Update

**DOI:** 10.3390/md13095920

**Published:** 2015-09-16

**Authors:** Janet Helen Fitton, Damien N. Stringer, Samuel S. Karpiniec

**Affiliations:** Marinova Pty Ltd., 249 Kennedy Drive, Cambridge, Tasmania 7170, Australia; E-Mails: Damien.stringer@marinova.com.au (D.N.S.); sam.karpiniec@marinova.com.au (S.S.K.)

**Keywords:** sulfated carbohydrate, immune modulation, anti-inflammatory, anti-cancer, anti-viral, anti-bacterial

## Abstract

Fucoidans are a class of sulfated fucose-rich polysaccharides found in brown marine algae and echinoderms. Fucoidans have an attractive array of bioactivities and potential applications including immune modulation, cancer inhibition, and pathogen inhibition. Research into fucoidan has continued to gain pace over the last few years and point towards potential therapeutic or adjunct roles. The source, extraction, characterization and detection of fucoidan is discussed.

## 1. Introduction

Fucoidans are a class of fucose-rich sulfated carbohydrates found in brown marine algae and echinoderms [1], and more recently identified in seagrasses [2]. In a previous review from 2011, potential applications of fucoidan were discussed [3]. In this update, we revisit some of the applications, discuss recent advances and focus on the characterization and sourcing of fucoidan. In particular, we will look at the therapeutic possibilities for fucoidan and fucoidan fractions, and which applications may prove to be clinically or commercially available. Other recent reviews in the field include those by Kwak [4], Pomin [5,6] Ale [7] and Ermakova [8].

Currently, fucoidans are available for use in cosmetics, functional foods, dietary supplements and for inclusion in pet, livestock and aquaculture feed supplements. To date there are no approved uses for fucoidan fractions in biomedical applications, either within biomaterials, or via direct administration (intravenous, intraperitoneal, intramuscular or subcutaneous). However, research into the use of fucoidan in drug delivery, biomaterials, as a topical agent and as an orally delivered agent for a variety of pathologies, appears promising. Whilst the class of molecules called “fucoidans” can be said to have broad similarities with regard to bioactivity, there are sometimes large differences [9]. For example, *Cladosiphon okamuranus* fucoidan did not inhibit breast cancer cell adhesion to platelets *in vitro*, whereas fucoidans from several other species including *Fucus vesiculosus*, inhibited that adhesion by more than 80% [9]. (Platelet adhesion is thought to be a mechanism for cancer cells to metastasise). Counter to this, oral *Cladosiphon* fractions attenuated tumour growth and increased survival in a mouse colon cancer model, an effect that was related to molecular weight profile of the fraction [10]. It is apparent that fucoidan fractions need to be standardized, assessed and validated for each particular application, and their main mechanism of action understood. As the research base expands, increased understanding about the specific, targeted activity of particular fractions can be properly exploited for therapeutic application.

## 2. Fucoidan Development as a Therapeutic

Despite the promising research publications in several potential areas of application, fucoidan has not yet been developed as a regulated therapeutic. There are a number of reasons for this. Firstly, fucoidan is a generic term for a class of moieties, and the research covers a large array of algal and echinoderm fucoidan source materials in addition to various extraction methods. To be suited to a regulated product, a fucoidan must be defined and reproducible. This can be achieved, but requires considerable resources and certainty over the source material availability over the long term. Sustainable, clean and regulated harvesting, or culturing of a single type of seaweed are required. Costs may be a limiting issue for fucoidans derived from expensive [11] and low yielding sources such as sea cucumbers (e.g., 0.025% from *Stichopus japonicas* [12]). “Patentability” is also important for companies interested in taking a product from a preclinical concept to clinical trials. Some notable recent patents cover the use of fucoidan in biomaterial scaffolds [13] and for treatment of clotting abnormalities [14].

In order to meet therapeutic regulatory requirements, the bioavailability, pharmacokinetic analysis and distribution of specific, characterized fucoidans needs to be considered, and the tools to enable these analyses are now becoming available. Conversely, the established safety [3,15] and availability of fucoidan as a food supplement or dietary supplement makes it widely available for “complementary” use. Accordingly, in the short term, most uses of fucoidan are likely to remain in this category. The field of enhancing bioactivity and controlling uptake of fucoidan with novel biomaterials is also being developed, and is discussed later in this review.

## 3. Bioavailability: Uptake and Distribution of Fucoidan

The subject of oral bioavailability still requires considerably more understanding. Earlier clinical research demonstrated the uptake of orally ingested *Undaria pinnatifida* fucoidan into serum [16] and of orally ingested *Cladosiphon* fucoidan in serum and urine [17]. The relative amounts of serum bioavailability, as assessed by antibody-based methods in these two studies, were very low. Since our last review there have been research papers demonstrating the oral uptake and fate of fucoidan. Nagamine *et al*., demonstrated the uptake and distribution of up to 2% w/w dietary *Cladosiphon* *okamuranus* fucoidan in a rat model, comparing normal absorption and nitrosamine-enhanced absorption [18]. Once again, whilst uptake was low, it was detectable. Histology using an antibody to the fucoidan indicated presence in the small intestine, jejunal epithelial cells, mononuclear cells in lamina propria and in sinusoidal non-parenchymal cells of the liver. In nitrosamine-enhancer-fed rats, fucoidan was found in Kupffer cells (specialised tissue macrophages that line blood vessels in the liver and function as scavengers, filtering out pathogens and raising immune responses) The same research group demonstrated transport of fucoidan across Caco2 cell monolayers *in vitro* and urinary excretion in a human after ingestion of *Cladosiphon okamuranus* fucoidan [19]. The fucoidan concentration was measured using a chromatography method, in which the fucoidan was excluded from the column and appears as an early peak. Using of a variety of transport inhibitors, the researchers showed that the fucoidan was most likely transported across the cell monolayer by active transport. Urinary excretion of fucoidan after oral ingestion of a fucoidan-containing beverage increased from the 3 h to 9 h time points. Further investigation of this research by other groups is justified. In the near future, pharmacokinetic studies on fucoidan will be greatly facilitated by these improved measurement techniques.

Oral drug delivery is often desirable, but not always possible, as actives can be destroyed by exposure to the acidic environment of the stomach. However, it has been found that chitosan nano-encapsulation of fucoidan exhibited a pH-dependent release mechanism [20]. This research showed that fucoidan inclusion into a nano-particulate chitosan composite was suitable for oral delivery of curcumin, releasing the active as pH increased. In another type of system, fucoidan nano-particles could be loaded with the cancer drug doxorubicin in a model system, and were more effective at inhibiting multidrug resistant cancer cells than the free drug [21]. The recent nano-particle technology has provided an appealing delivery platform for fucoidan *in vivo*. Fucoidan in liposomes was found to be more effective than simple fucoidan in an oral delivery mouse model of xenografted osteosarcoma [22]. The increased efficacy of the liposome fucoidan was proposed to be due to increased uptake.

Future research into increasing the efficacy and/or bioavailablity of fucoidan will increase the utility of fucoidan as a therapeutic.

## 4. Comparison between Types of Fucoidan

In the 2011 review [3], we outlined biological response differences between fucoidans derived from different species, and between high or low molecular weight fractions of fucoidan derived from the same species. Recent research further underlines the differences between fucoidan sources in terms of biological response, emphasizing that any potential therapeutic application requires a specific, validated assessment of a characterised fucoidan. In a 2015 paper, Zhang *et al*., investigated four types of fucoidan derived from *Ascophyllum nodosum*, *Fucus vesiculosus*, *Macrocystis pyrifera* and *Undaria pinnatifida*, the latter two now also available from the research chemical supplier Sigma-Aldrich [23]. These fucoidans were assessed for effects on apoptosis of neutrophils, activation of natural killer (NK) cells, maturation of dendritic cells, proliferation and activation of T cells, and adjuvant effect *in vivo*. Fucoidans from *Macrocystis pyrifera* and *Undaria pinnatifida* strongly delayed human neutrophil apoptosis at lower concentrations (10–20 micrograms/mL) than those from *Ascophyllum nodosum* and *Fucus vesiculosus* (50–100 micrograms/mL) *Macrocystis pyrifera* fucoidan especially promoted NK cell activation and cytotoxic activity against target (YAC-1) cells. In addition, *Macrocystis pyrifera* fucoidan induced the strongest activation of spleen dendritic cells and T cells and ovalbumin specific immune responses compared to other fucoidans. The authors suggested that fucoidan from *Macrocystis pyrifera* may have most potential as a therapeutic agent or as a vaccine adjuvant. Adjuvants can boost responses to vaccines. Interestingly, a Japanese study showed that orally ingested *Undaria* fucoidan at a 300 mg/day was effective in raising an antibody response to influenza vaccines in elderly subjects [24], although in the Zhang *et al*., study, *Undaria* fucoidan was the only fucoidan tested that did not raise cytokine responses when administered *iv* to mice. This may reflect differences in the fucoidan preparations between the studies, or that the adjuvant activity is dependent on another pathway. The composition of the various fucoidans was analysed during the Zhang *et al*., study, showing no clear structural rationale for the differences in biological effect. There was no direct correlation with sulfation levels, however the uronic acid-containing fucoidans (*Macrocystis pyrifera* and *Ascophyllum* *nodosum*) were both more effective in activating dendritic cell responses and this provides a direction for future refinement of this activity Further on in this review we discuss the Toll like receptor (TLR) stimulating activity of fucoidans [25], which is a known route to effective adjuvant activity [26].

A comparative anticoagulant study of various molecular weights of fucoidan from *Laminaria* *japonica* found that anticoagulant activity in the global clotting assay, activated partial thromboplastin time (aPTT), depended on the molecular weight and also on the molar ratio of fucose to galactose [27]. Following earlier work on the potential of fucoidan to “bridge” a gap in defective clotting cascades, *Fucus vesiculosus* fucoidan fractions were shown to have a minimum size requirement to exert a “procoagulant effect” in clotting deficient blood [28]. Clement had previously determined that particular structures, rather than molecular weight or individual sugars, affected the anti-complementary (C4) activity of fucoidan [29] and this may also be the case for other biological activities. In summary, fucoidan fractions require screening for activity to optimise any particular bioactivity and while activity is not necessarily linked to sulfation, sugar content or molecular weight, useful trend data can be found.

## 5. Characterization: Source, Extraction and Analysis

Fucoidans, like most polymers, are polydisperse. This means that the polysaccharide is not well described by a discrete molecular weight, but is in fact a mixture of molecular weights. The molecular weights are often quoted as a range, or with an “average” or median peak weight (see Table 1 and Table 2 below). This mixture of similar, but different molecular weight molecules can be additionally complex, as fucoidans extracted from seaweeds are branched molecules. Thus, as similar molecular weight molecules may have slightly different branching patterns, perhaps as a result of the original polysaccharide being hydrolysed during extraction, the variety of molecules present—even for a discrete molecular weight—can be large. Note that fucoidan from echinoderms is not branched, but linear, as demonstrated by Pereira and Mulloy [30]. Mulloy had earlier demonstrated that echinoderm fucoidans have a regular repeat tetrasaccharide motif [31].

Fucoidans also display distinct structural motifs, dependent on the species from which they are extracted. For instance, hydrolysed fucoidan extracts from the *Fucaceae* family typically yield high levels of fucose and low levels of other constituent monosaccharides. Contrasting this structure is the fucoidan isolated from *Undaria pinnatifida*, of the *Alariales* family, which contains significant amounts of galactose in its carbohydrate backbone [32]. Such species variations are also evident in the sulfate and acetyl content of fucoidans, further adding to the difficulty of elucidating structure-function relationships for these compounds. It appears that uronic acid components, either attached to the fucoidan or as contaminants, may be associated with biological responses [23].

**Table 1:** Absolute mass percentages of components of normalised fucoidan extracts from varying species determined using standardised methods by Marinova Pty Ltd.

Species	Fucose (%)	Xylose (%)	Galactose (%)	Arabinose (%)	Rhamnose (%)	Uronic Acid (%)	Sulfate (%)	Cations (%)	Sulfation Ratio
*Fucus vesiculosus*	43.1	8.8	2.2	1.2	0.2	8.7	30.1	5.7	0.81
Sigma Crude *Fucus vesiculosus*	45.9	3.3	4.3	0.0	0.0	7.0	32.0	7.6	0.92
*Macrocystis pyrifera*	30.5	2.2	5.6	0.0	1.7	12.4	32.4	15.1	1.1
*Cladosiphon* sp.	51.2	2.1	1.3	0.0	0.0	15.5	23.0	7.0	0.58
*Laminaria japonica*	34.1	1.0	4.2	0.3	1.0	14.4	31.7	13.2	1.0
*Ecklonia radiata*	19.0	11.0	12.0	6.2	1.7	25.5	19.1	5.4	0.45
*Durvillaea potatorum*	27.9	2.1	6.2	0.3	0.7	32.4	21.4	8.9	0.57
*Lessonia nigrescens*	26.2	8.1	13.0	2.0	0.9	12.9	29.1	7.8	0.82
*Alaria esculenta*	37.5	3.4	16.4	0.6	1.3	12.3	20.2	8.2	0.50
*Undaria pinnatifida*	32.6	0.0	25.2	0.5	0.4	4.0	29.6	7.7	0.85
*Pelvetia canaliculata*	38.3	3.0	5.6	0.1	0.0	4.3	43.0	5.7	1.4

**Table 2:** Molecular weight profiles of fucoidan extracts determined by Gel Permeation Chromatography, with the aid of a Size-Exclusion Column, and are reported relative to dextran standards using standardised methods by Marinova Pty Ltd.

Species	Peak Average MW (kDa)
*Fucus vesiculosus*	82.5
Sigma (crude) *Fucus vesiculosus*	20.7
*Macrocystis pyrifera*	176.4
*Cladosiphon* sp.	1927.2
*Laminaria japonica*	395.4
*Ecklonia radiata*	528.2
*Durvillaea potatorum*	336.3
*Lessonia nigrescens*	491.8
*Alaria esculenta*	147.9
*Undaria pinnatifida*	51.7
*Pelvetia canaliculata*	103.9

Fucoidan can be extracted and purified from the original seaweed by a number of processes. Laboratory methods have traditionally relied on acidic extraction in aqueous media using heat, whereupon the fucoidan is released into the water phase. Ethanol (up to 60% *v/v*) is then used to precipitate the fucoidan, relying on the inherent lack of solubility of the molecule in less polar solvents. The resulting precipitate can then be further solubilized and re-precipitated if additional purification is required. The 1952 descriptions by Black remain an excellent guide to these classical methods [33].

Newer methods of preparation include enzyme mediated release and microwave assisted extraction (MAE). Endo- and exo-enzymes derived from either marine bacteria or marine molluscs have the ability to digest fucoidan and thus aid in their extraction. In one study, fucoidan hydrolase, alpha-l-fucosidase, and arylsulfatase from the marine mollusc *Littorina kurila* were isolated and described [34]. It was found that alpha-l-fucosidase and arylsulfatase (synthetic substrates) cannot hydrolyze natural fucoidan, whereas fucoidan hydrolase cleaves fucoidan to produce sulfated oligosaccharides and fucose. More recently, Silchenko *et al*., isolated an enzyme from a marine bacteria with specific hydrolytic activity for fucoidans extracted from *Fucus evanescens* and *Fucus vesiculosus*, which was not replicated in fucoidan from *Saccharina cichorioides* [35]. Terrestrial fungal enzymes that degrade *Laminaria japonica* fucoidan were screened and one from *Asperigillus niger* found to be a strong contender for fucoidanase production by Rodriguez-Jasso *et al.* [36]. Such specificity hints at the unique bioactivities afforded by fucoidans extracted from different species of algae. To date, no fucoidanases are available at a commercial scale for fucoidan processing from marine algae.

Microwave techniques currently offer laboratory-scale isolation of fucoidans with short extraction times. In future, this technology might be used commercially. In a recent study the structural features of fucoidan extracts from *Fucus vesiculosus* were found to be influenced by the extraction pressure, time and water: algal ratio [37]. Such structural variations resulting from extraction methods have been well described by Ale *et al.* [7], who reinforce the importance of maintaining structural integrity throughout the fucoidan extraction process in order to achieve consistent biological function. When compared to classical extraction, microwave assisted extraction reduced the *in vitro* anticancer activity of fucoidan fractions from *Undaria pinnatifida* [38]. However, a microwave assisted extract of fucoidan from *Ascophyllum nodosum* was effective in yielding “antioxidant” fractions [39].

The abundance of isolation techniques for fucoidan leads to a discussion on the characterization of these extracts. Without appropriate extraction and purification controls, fucoidan extracts may contain significant quantities of non-fucoidan components, thus any inference of the extracts’ biological activity is fraught with uncertainty. This is particularly true when comparing biological activities of extracts from laboratory-based methods which have not been validated. In the last decade, the availability of commercial scale, validated fucoidan extracts has allowed a more systematic approach to fucoidan research. Scale-up from the laboratory requires consideration of volumes, residual solvents, chemical and microbiological safety. All of these considerations may alter the chosen method of isolation.

The characterization of fucoidan extracts has advanced in recent years as appropriate chemical analytical techniques have been developed. Such techniques allow for comprehensive carbohydrate profiling and linkage analysis together with sulfate, acetylation and counterion quantification. Together, these analyses provide the data necessary to adequately describe and contrast fucoidan extracts from different sources and species. Examples of such differences in composition are summarised in Table 1 below, for fucoidan extracts produced systematically using an aqueous extraction process under controlled pH, temperature and time conditions. The data presented here was prepared for this review at Marinova Pty Ltd.’s laboratory according to the methods described next.

The carbohydrate profile was determined using a GC-based method for the accurate determination of individual monosaccharide ratios in a sample. This method relies on the preparation of acetylated alditol derivatives of the hydrolysed samples [40]. The uronic acid content was determined by spectrophotometric analysis of the hydrolysed compound in the presence of 3-phenylphenol, based on a method described by Filisetti-Cozzi and Carpita [41]. Sulfate content was analysed spectrophotometrically using a BaSO_4_ precipitation method (BaCl_2_ in gelatin), based on the work of Dodgson [42,43]. Cations, including Na, K, Ca and Mg, were determined by Flame Atomic Absorption Spectroscopy.

The crude *Fucus vesiculosus* extract historically provided by Sigma-Aldrich, St. Louis, MO, USA, (Code F5631) was also analysed and the composition of its fucoidan component is also provided for comparative purposes. In addition to the fucoidan components described below, the Sigma-Aldrich extract (Code F5631) also contained 3.9% polyphenols, as determined by the Folin-Ciocalteu reagent [44] and 0.4% mannitol.

Note that for the purposes of the fucoidan compositions in Table 1, the uronic acid content (as determined spectrophotometrically against glucuronic acid standards), has been included in the fucoidan composition. Whilst the inclusion of uronates in fucoidans from *Cladosiphon* sp. has been clearly established [45], caution is urged in assuming that the full uronic acid component determined below is contained within the fucoidan polymer. Further linkage analyses would be required to fully distinguish fucoidan uronates from co-extracted alginate uronates.

Variations in fucoidan composition with respect to its source species are clearly highlighted in Table 1, with extremes in fucose content from 51.2% for *Cladosiphon* sp. to 19.0% for *Ecklonia radiata*. Similarly, galactose content varies from the widely recognized galactose-rich *alariales* *Undaria pinnatifida* and *Alaria esculenta* at 25.2% and 16.4% down to the low galactose-containing *Cladosiphon* sp. and *Fucus vesiculosus* at 1.3% and 2.2% respectively. Broad ranges in sulfation ratio are also observed, from lows of 0.50 for *Alaria esculenta* to highly sulfated *Pelvetia canaliculata* at 1.4. Whilst not analyzed for all samples in the above sample-set, fucoidan acetyl content is also known to vary substantially with, for example, as much as 3% by mass acetyl content in *Undaria pinnatifida* fucoidan and less than 0.5% for *Fucus vesiculosus* fucoidan, as determined by Nuclear Magnetic Resonance (NMR) spectroscopy techniques (unpublished data). With such complex compositional variations observed between species that are exposed to identical extraction and purification procedures, it is evident that comprehensive chemical analyses are required for any exploration of fucoidan bioactivity.

Additional methods of examination of the tertiary structure of fucoidans include NMR spectroscopy and mass spectroscopy. As recently outlined by Anastyuk *et al.*, mass spectroscopy has limitations because the fucoidans tend to desulfate rather than ionize during measurement [46,47]. They describe rational examination of different fucoidans using exhaustive hydrolysis followed by MALDI-TOF and ESI mass spectroscopy.

As discussed previously, the molecular weight profiles of fucoidan extracts are polydisperse, and can be highly dependent on the extraction methodology used. This can be seen in the contrasting molecular weights of the aqueous *Fucus vesiculosus* extract reported here, and that of the crude *Fucus* *vesiculosus* extract available from Sigma-Aldrich, which exhibits a markedly lower molecular weight profile, perhaps demonstrating significant hydrolysis of the fucoidan during processing. The molecular weight peak averages of the extracts characterized in Table 1 are summarized below in Table 2.

Further to the characterization of the fucoidan itself, the presence of other bioactive compounds should also be established as they will interfere with any structure-function study. These actives may themselves be complex carbohydrates such as the ubiquitous alginates, or the glucose-rich storage polymers prevalent in the laminariales, such as laminaran [48]. Additionally, substantial quantities of non-polysaccharide components, such as the sugar-alcohol, mannitol, and importantly, potent marine antioxidants—polyphloroglucinols—are also routinely observed in fucoidan-containing extracts [49,50]. The presence of these components should be recognized for the contributing effect they may have on biological activity such as antioxidant activity. Analysis requires quantification using appropriate methods such as the spectrophotometric determination of polyphenols by the Folin-Ciocalteu reagent [44,51,52].

## 6. Methods for Quantifying and Identifying Fucoidan in Biological Fluids

A fundamental requirement for understanding the pharmacokinetics of fucoidan is the development of robust analytical methods. Methods for characterizing fucoidan include chemical and structural analysis as discussed above. Biologically, identifying the presence of fucoidan in fluids or in histology sections requires different approaches, and has been achieved by chromatography techniques, antibody labeling [16,17,53], dye methods [54] and most recently, electrochemical detection [55].

Thus far, monoclonal antibodies have been used to identify fucoidan from different species in marine algae, and in samples from animal studies and human clinical samples [3]. There are limitations to the antibody recognition of fucoidan, as it is unclear whether all sub-fractions of a fucoidan-heterodisperse preparation are antibody labeled. The specificity of antibodies may also be an issue, as cross-reaction with heparin in a sample is also common (unpublished observations). However, antibody techniques remain flexible and especially useful in staining tissue sections [18]. When validated against an absolute chemically-defined standard, antibody-based detection in microplate assays will prove extremely useful.

The recent work by Nagamine *et al.* [19] uses a chromatography method in which size exclusion columns are used, along with UV absorbance detection, to analyze samples. The peaks observed as the fucoidan elutes from the column can differentiate it from other compounds, providing the components of the biological system are known. However, there was no specific sugar identification of the fucoidan in the urine sample in the study.

Lee *et al*., reported on a low cost and easily available methylene blue dye-based method for sulfated polysaccharides from *Hizikia fusiformis* immobilized onto filter paper [54]. The bound dye could be eluted and measured by absorbance, or even measured *in situ* on the filter paper. This method allowed for selective detection of fucoidan at low levels (1–20 micrograms) in the presence of interfering compounds such as alginic acid.

An electrochemical method for fucoidan detection was reported by Kim [55] where ion-sensitive polymer membranes were developed to quantity and differentiate fucoidans from different sources in solution. The sensitivity of the method was good, analyzing down to nanogram quantities in solution, including serum and urine. This method offers the potential for rapid fucoidan identification and quantification in biological fluids, with the ability to differentiate from endogenous molecules such as heparin.

The development of these methods means that researchers now have a “toolkit”. Use of the methylene blue method, and the electrochemical method allows for optimization of extraction methods in the lab, and will help to analyse and differentiate “unknown” samples. Further development of the electrochemical method and size exclusion chromatography shows great promise in the analysis of clinical samples from fucoidan trials, which will in turn, help to establish the pharmacokinetics of fucoidan.

## 7. Cancer and Fucoidan

There has been continuing interest in the use of fucoidan as an anti-cancer agent. An excellent review which can be used for general reference was presented by Kwak in 2014 [4]. Proposed cellular mechanisms for “anti-cancer” activity of fucoidan were also recently reviewed [56]. Fucoidan is unlikely to be used as a sole agent for cancer treatment where there are known effective therapies, yet as a non-toxic edible product it is an easily deliverable active. Here, we will discuss research into the potential for orally delivered fucoidan as an “adjunct” therapy to conventional treatment. Fucoidan may find a role either to reduce side effects, to enhance the therapeutic effects of conventional therapy, or to address cancer for which there are no known therapy options.

Kwak covers the main mechanisms by which fucoidan could act to inhibit cancer: scavenger receptor modulation; immune activation; anti-angiogenesis; the blockade of metastasis; mobilisation of stem cells and interference with SDF1/CXCR4 axis; anti-oxidant and pro-oxidant effects. As Kwak notes, the anti-cancer activity of fucoidan is likely to be via more than one single pathway [4].

The direct effects fucoidan on cancer cells are often via a nuclear factor kappa-light-chain-enhancer of activated B cells (NF-κB) pathway, mediated by PI3K/Akt and ERK signaling pathways [56]. More recent research indicates that fucoidan may induce apoptosis in breast and colon cancer cells via modulation of the endoplasmic reticulum stress cascades [57]. Co-cultured cell experiments, and then whole-animal experiments have also illustrated “anti-cancer” activities of fucoidan preparations from the standpoint of immune clearance of cancer cells, as described in the review by Kwak [4].

Perhaps the first issue for any therapeutic is bioavailability (addressed earlier in this review). If fucoidan is serum bioavailable, and has favorable pharmacokinetics, there may be a role as a cancer therapeutic. To date, there is doubt as to whether serum levels of dietary fucoidans would reach the levels necessary to directly inhibit cancer cell growth. At first, it may seem obvious that direct systemic effects- such as inhibition of tumour growth [10]—must be due to serum uptake of fucoidan. However, it may be the case that fucoidan exerts its effects via the gut, either by modulating immune cells directly, or secondarily, by altering the balance of microbiota.

More easily accessible cancers—such as those occurring within the gastrointestinal tract—could be exposed to orally delivered fucoidan without the need to consider uptake. Fucoidan may have utility as a sole agent, or adjunct agent, to delay or prevent the initiation of cancer. Other natural products have been assessed in colon cancer models, and this preventative approach can provide a route to developing a “functional food” for healthy colon [58].

One possible application is the use of fucoidan as an agent to reduce side-effects such as gastrointestinal mucosits during chemotherapy. A preparation of fucoidan (50–500 kDa), derived from the sea cucumber *Acaudina molpadioides*, was applied in a model of cyclophosphamide-induced intestinal mucositis in mice [59]. Cytokine levels were moderated, and the preparation was highly effective in restoring mucosal IgA. The common chemotherapy drug Cisplatin causes delayed motility, decreases in body weight and changes in the levels of gastric hormones gastrin and serotonin. Low molecular weight fucoidan prevented these changes in a manner similar to the commonly used drug, Ondansetron, suggesting that fucoidan can assist in maintaining normal gastrointestinal function during chemotherapy [60]. The effects in these chemotherapy-induced models is echoed in recent work showing that oral fucoidan (*Fucus vesiculosus*) was effective in reducing inflammation in a colitis model [61].

Secondly, fucoidan could be useful as an adjunct oral therapy during or after conventional chemotherapy or radiotherapy. Several studies, using *in vitro* models, have noted potential synergies with chemotherapy, as outlined in Table 3. However, as noted in cancer cell lines expressing EGFR receptors, the effects could also be antagonistic [62]. Zhang *et al.*, using *Cladosiphon* fucoidan fractions, showed synergistic effects with three chemotherapy agents in two breast cancer cell lines [63]. In yet another type of cancer, human malignant lymphoid cell lines were pretreated with fucoidan prior to treatment with the chemotherapy drug etoposide, greatly increasing its efficacy [64].

**Table 3:** Potential synergies with chemotherapy in *in vitro* and *in vivo* models.

Fucoidan Source	Chemotherapy	Model	Outcome	Concentration	Reference
*Fucus evanescens*	Etoposide	Human malignant lymphoid cell lines, MT-4 and Namalwa	Enhanced etoposide-induced caspase dependent cell death pathway in MT-4 but not Namalwa cell line	Presensitisation with 500 micrograms/mL fucoidan, prior to etopside treatment	Philchenkov *et al.* [64]
*Cladosiphon* sp. prepared by enzymatic digestion	Cisplatin, tamoxifen or paclitaxel	MDA-MB-231 and MCF7 breast cancer cells.	Down-regulation Bcl-xL Mcl-1. Decreased phosphorylation of ERK and Akt in MDA cells, increased phosphorylation of ERK in MCF-7 cells. Increased intracellular ROS, reduced glutathione (GSH)	Fucoidan extract at 200 and 400 micrograms/mL concurrently with chemotherapy drugs	Zhang *et al.* [63]
Unknown source or composition fucoidan preparation	Lapatinib	EGFR/ERBB2-amplified cancer cell lines	Synergistically inhibit OE33 but antagonized in ESO26, NCI-N87, and OE19	10 to 1000 micrograms/mL	Oh *et al.* [62]

These early results are interesting, but require further cancer-specific, fraction-specific investigation, as well as translation into *in vivo* models.

Clinical studies in Japan have noted that ingestion of 4.05 g per day of fucoidan (*Cladosiphon okamuranus*) reduced the clinical toxicity indicator “fatigue” in a small cohort of subjects (10) undergoing oxaliplatin plus 5-fluorouracil/leucovorin or irinotecan plus 5-fluo-rouracil/leucovorin chemotherapy for unresectable colon cancer, compared to those not taking fucoidan [65]. Patients taking fucoidan were able to tolerate more rounds of chemotherapy. At this stage it is unclear why this might be the case, but the observations warrant follow-up studies. Fatigue during exercise was investigated in a mouse model, and found to be reduced by oral *Laminaria japonica* fucoidan at an equivalent human dose of 1.5 g per day [66]. In the model, the reduced fatigue was associated with decreased serum lactate and ammonia, increased serum glucose and lower serum triglyceride levels.

In a recent Australian interaction study, the effects of fucoidan on the drug levels in the serum of subjects with breast cancer were monitored, for patients taking the common hormone drugs tamoxifen or letrozole. There was no significant interaction with these long term treatments, showing that fucoidan at 1 g per day was safe [67]. Any proposed use for fucoidan as an adjunct to chemotherapy should ensure that it does not interfere with serum pharmacokinetics of the chemotherapy drugs. This provides reassurance to patients and physicians.

Amongst the many variations in fucoidans, and variations in cancer types, there appear to be broad patterns for mechanisms of anti-cancer activity. Not all cancers will respond in the same way at the direct cellular level, yet cancer inhibitory effects may be mediated *in vivo* via immune stimulation or anti-metastatic mechanisms.

Fucoidan is known to interfere with the binding of a cellular receptor called “CXCR4” to a chemokine known as “CXCL12” or “stromal derived factor 1”, chiefly by binding to CXCL12 [68,69]. The binding of these receptors can be critical for the establishment, growth and metastasis of tumors, so prevention of binding may assist in inhibiting cancer. Since Kwak’s review which also discussed this subject, fucoidan from *Saccharina latissimi* and *Fucus vesiculosus* and heparin were investigated for their effects on human Burkitt’s lymphoma cells [70]. The fucoidan fractions bound CXCL12, and thus prevented CXCR4 binding and the downstream effects such as secretion of matrix-degrading enzymes necessary for metastasis. In this case, the higher molecular weight, unfractioned fucoidan was more effective than the smaller sub-fractions, once again illustrating subtle differences in biological activity. Fucoidan may lessen the effects of acute pancreatitis (inflammation rather than cancer), possibly as a result of the ability of fucoidan to block selectins [71]. The effects of fucoidan from the lesser known brown algae, *Turbinaria conoides*, have been studied *in vitro* using a specific pancreatic cancer cell line. The research indicated that fucoidan decreased the ability of cancer cells to secrete the matrix-degrading enzymes they required to metastasize and spread [72]. Interestingly, pancreatic cancer is also highly dependent on CXCR4 to spread. Recent research showed that the CXCR4 inhibitor plerixafor was effective in blocking the metastatic potential of pancreatic cancer in a mouse model [73], indicating once more a potential role for fucoidan fractions to be investigated in this application.

In a Lewis lung cancer model in mice, oral *Fucus vesiculosus* fucoidan lessened body weight loss and reduced lung masses [74]. Unusually, the fucoidan was pretreated with simulated gastric and intestinal juices prior to administration. In this comprehensive study, fucoidan down-regulated the expression of a number of key markers associated with tumor development, spread and proliferation. These markers included matrix metalloproteinases, NF-κB and vascular endothelial growth factor (VEGF). The focus on VEGF reflects a current interest in drugs that block the spread of cancers expressing this marker, such as the antibody drug bevacizumab, used extensively in advanced cancers to block angiogenesis [75]. In this study, fucoidan showed promise as chemo-preventative agent for minimizing weight loss symptoms and reducing tumor proliferation. The ability of fucoidan to reduce expression of VEGF is also displayed in recent research into macular degeneration [76]. Fucoidan reduced VEGF expression in retinal pigment epithelial cells, and the effects were additive to bevacizumab, showing that fucoidan could be considered as an adjunct agent.

Survival rates are low for many types of liver cancer—including poorly differentiated hepatocellular carcinoma. Recent research investigated the effects of fucoidan on cells from this cancer type *in vitro*. The researchers studied the effects of fucoidan on a proliferation regulator known as AMP-activated protein kinase (AMPK), as well as its downstream metabolism and cell cycle-related molecules, in a poorly differentiated human hepatoma HLF cell line. The results suggested that fucoidan inhibited proliferation of the cancer cells via the AMPK-associated suppression of fatty acid synthesis and cell cycle G1/S transition [77]. This data is reflected by several other papers describing effects of fucoidan on normal and carcinoma-like hepatocellular lines including HepG2 [78].

Oral cancers present treatment challenges, and may be accessible to localised application of fucoidan. Fucoidan causes apoptosis in a particular type of oral cancer called “mucoepidermoid carcinoma” [79]. Fucoidan decreased cell proliferation and induced caspase-dependent apoptosis via down-regulation of phosphorylation of the extracellular signal-regulated kinase ERK1/2. Fucoidan holds promise as a therapeutic agent for treatment of this type of oral cancer and should be investigated further.

Lastly, prostate cancer is a globally significant disease and one of the most common causes of cancer death in men. In a new Korean study, the anti-cancer effect of fucoidan from *Undaria pinnatifida* was assessed *in vitro* using human prostate cancer cells (PC-3). Fucoidan was shown to induce apoptosis of cancerous PC-3 cells in a manner that suggested fucoidan could induce both “intrinsic” and “extrinsic” apoptosis pathways. The results of this study offer encouraging prospects for the potential of fucoidan in the treatment of prostate cancer if bioavailability and delivery can be addressed successfully.

In summary, fucoidan may offer promise as a complementary medicine to alleviate some of the side effects of chemotherapy. Activity as a sole agent, or adjunct agent to chemotherapy is interesting, but will require more development for use as a regulated therapeutic.

## 8. Immune Modulation

Modulation of immunity by fucoidan can be used as a tool to disrupt disease processes, including cancer and pathogen infections. Perhaps the most compelling research in immune modulation showed that ingestion of 1 g per day of *Undaria pinnatifida* fucoidan assisted the antibody response to seasonal vaccines in a group of very elderly rest-home residents [24]. This minor intervention is equivalent to that induced by the ingestion of the recommended dietary intake of fresh fruit and vegetables [80]. However, in challenging health conditions and extreme old age, dietary intake may not meet recommendations, whereas 1 g daily of fucoidan can easily be administered.

Dendritic cells are a class of cells that direct immune function within tissues which include, for example, the Langerhans cells of skin and mucosa. They act as a type of “conductor” of immunity, presenting antigens to other immune cells and starting the process of recognizing pathogens and initiating a specific response. *Fucus vesiculosus* fucoidan’s effects on the function of spleen dendritic cells, as well as its adjuvant effect, were investigated in an elegant series of experiments *in vivo* and *in vitro* [81]. The results suggested that fucoidan does function as an adjuvant, enhancing an adaptive immune response and activating cytotoxic T cells. Further research from the same group investigated fucoidan from the *Ascophyllum nodosum* species of seaweed and demonstrated its ability to induce dendritic cell maturation and enhance immune response *in vitro* and *in vivo* [82]. The results reinforce that fucoidan could be developed for novel therapeutic strategies to combat infectious diseases and cancer.

Toll like receptors (TLRs) are a class of membrane spanning receptors that are a key part of the innate immune system. Researchers demonstrated that fucoidans from *Laminaria japonica*, *Laminaria cichorioides* and *Fucus evanescens* activate and bind to “toll-like receptors” in human cells [25]. Fucoidans interacted with TLR-2 and TLR-4, but not with TLR-5, and were non-toxic for the cells. Binding to TLR activated the mediator NF-κB to stimulate immune defences. Fucoidan treatment resulted in increases in cytokines, chemokines and the expression of MHC molecules, demonstrating the activity of both adaptive and innate immune cells. The study established that fucoidan protects cells from pathogens by encouraging both innate and specific immune responses.

Immune modulation is one of the most promising areas for orally delivered fucoidan, available currently in “dietary supplement” format. The ability to enhance immune response to vaccine or pathogen epitopes is desirable and achievable. The use of fucoidan in a clinical setting to maximize particular therapeutics will require more work, with the development of oral delivery offering perhaps the easiest route to a commercially available product.

## 9. Anti-Pathogen

### 9.1. Leishmaniasis

In the last review, we covered a number of anti-pathogenic qualities of fucoidan. One of the key findings was a remarkable clearance of the protozoan pathogen Leishmania in a mouse model using orally delivered fucoidan [83]. In 2014 the same research group demonstrated that the Leishmania parasites inhibit induction of the MAPK pathway in macrophages, and prevent the production of nitric oxide and reactive oxygen species that would destroy them [84]. Fucoidan reactivates this mechanism, and thereby allows clearance of the parasites.

### 9.2. Viruses

Influenza A infection by different subtypes (H5N3 and H7N2) was modulated by oral ingestion of a low molecular weight *Undaria pinnatifida* fucoidan fraction (9 kDa) in mouse models [85,86], building on earlier research showing the additive effects of fucoidan on the common influenza drug oseltamivir [87]. As discussed in this paper, the structure of fucoidan is complex, and cannot be completely described by simple chemical methods. Using FTIR, FT-Raman and NMR spectroscopy in addition to methylation analysis, the researchers demonstrated that the active fraction was an *O*-acetylated sulfated fucogalactan.

Canine distemper is a condition caused by a paramyxovirus type virus that affects a wide variety of animals. In a recent study, *Cladosiphon* fucoidan at extremely low levels inhibited cellular entry of the canine distemper virus [88] indicating potential for a therapeutic role in veterinary and animal health applications.

Newcastle disease is a serious, contagious viral disease affecting birds, particularly poultry, and can result in significant economic loss to breeders and growers. Fucoidan demonstrated elevated antiviral activity against one of the most prevalent strains of Newcastle disease, while exhibiting no toxicity. Consistent with other research in this area, these studies confirmed that fucoidan provided excellent protection against viral cellular penetration [89]. There may be potential for fucoidan to be used as low-toxicity antiviral compound in the poultry industry as a preventative measure against infection by Newcastle disease, although this requires challenge testing in whole animals.

### 9.3. Anti-Bacterial Effects

In general, fucoidan is not thought to have “direct” anti-bacterial effects. However, in a culture system, fucoidan was found to strongly increase the effectiveness of antibiotics against oral bacteria [90], indicating a promising avenue for reducing antibiotic use levels in oral infections. Resistance to bacterial infection might be increased by ingestion of fucoidan, for example: In a *Caenorhabditis elegans* model of *Pseudomonas* infection, addition of a fucoidan-rich extract of *Ascophylum nodosum* greatly increased survival rates of the test organism, and decreased the biofilm formation and motility of *Pseudomonas*. Genes for innate immunity were strongly upregulated and conversely, the bacterial genes for virulence factors, toxic metabolites and quorum sensing were down regulated [91].

*Helicobacter pylorus*, the causative agent of stomach ulcers, is successfully inhibited by fucoidan. This subject has recently been reviewed by Besendnova [92].

### 9.4. Endotoxins

Endotoxins (lipopolysaccharides) created by Gram negative pathogenic bacteria are the means by which these bacteria cause tissue damage. In recent years, the presence of serum lipopolysaccharide either from dietary intake, sub-chronic infection or alterations in gut microbiota has been found to correlate to perturbations in lipid metabolism and in cardiovascular disorders [93]. The western diet may contribute to the weakened mechanism of endotoxin detoxification [94] giving potential for the ingestion of fucoidan to protect against the effects of endotoxin. Orally or subcutaneously delivered fucoidan was strongly protective in an endotoxin affected mouse model [95]. If orally delivered fucoidan can reduce endotoxin induced metabolic damage in a clinical setting, it would be a highly desirable dietary supplement.

## 10. Fucoidan as an Anti-Inflammatory Agent

A key property of fucoidan is “selectin blocking” [3]. These adhesion molecules are found on platelets (P-selectins), and leukocytes (L-selectins). Blocking the action of selectins can attenuate inflammation by preventing the passage of inflammatory cells into tissue spaces. It can also prevent the adhesion of platelets. As described in the last review, previous research has shown that fucoidan can be used effectively in animal models to prevent post ischemic inflammatory damage [3]. Here we describe some of the recent research into possible applications of fucoidan as an anti-inflammatory agent.

Over 5% of elderly men experience aortic abdominal aneurysm—a localised widening of the main artery from the heart as it passes through the abdomen. The rupture of this aneurysm can be fatal and usually requires emergency surgery. Aneurysms are partly an inflammatory condition. In an animal model involving a bacterial-stimulated blood clot in the aneurysm, fucoidan was shown to lessen the swelling of the artery by reducing the number of inflammatory neutrophils entering the area. Immunohistological analysis showed a substantially less P-selectin immunostaining at the luminal surface of aneurysms in fucoidan-treated rats compared to the other groups, suggesting an interaction between fucoidan and P-selectin. It appears that fucoidan, if further developed, could present a therapeutic option in the treatment of abdominal aortic aneurysm [96].

Excessive numbers of these selectin receptors are found in most acute inflammatory conditions, including pancreatitis (a painful inflammation of the pancreas that can lead to cancer). Unfortunately, there are few treatment options for pancreatitis, and new treatment options are badly needed [97]. Carvalho *et al*., showed that intravenous delivery of 25 mg/kg fucoidan from *Fucus vesiculosus* reduced the severity of acute pancreatitis in mice, suggesting potential for a new and effective therapeutic approach for this debilitating condition [71]. In this research, fucoidan significantly decreased the higher levels of enzymes in mice exhibiting acute pancreatitis, while mice administered the fucoidan also had fewer detrimental changes to the structure of the diseased pancreas. Furthermore, fucoidan was shown to inhibit neutrophil infiltration and reduce the levels of pro-inflammatory cytokines.

Topical application of fucoidan can also, despite the usually high molecular weight, exert an anti-inflammatory effect on inflamed skin. In cosmetic research, a 0.3% w/w formulation containing either *Undaria pinnatifida* or *Fucus vesiculosus* fucoidan was highly effective at inhibiting the erythema and water loss caused by UV-induced inflammation [98]. Japanese researchers demonstrated that fucoidan mediated the suppression of IgE in blood cells from patients with atopic dermatitis [99]. In addition, application of *Undaria pinnatifida*-derived fucoidan to a mouse model of allergic dermatitis was shown to be as effective as dexamethasone [100], the commonly used corticosteroid treatment, which has significant drawbacks for long term use. This research paves the way for fucoidan to be incorporated into dermatological formulations and other topical products targeting inflammatory skin conditions.

Fucoidan is well suited as a targeted treatment for gut inflammation. Recent research demonstrates the profound inhibitory effect of orally delivered fucoidan in a mouse model of colitis [61]. Fucoidan from *Fucus vesiculosus* was given daily at a 1 g human dose equivalent. Clinical signs, histological scores and cytokine levels were all significantly lowered as compared to the controls. Cumulative histological disease scores for the distal colon were reduced by up to 36.3% by fucoidan extracts, while weight loss—a common and often undesirable side effect of colitis—was reduced by more than 50%. The capacity of a modest dose of fucoidan to reduce inflammation and retain epithelial integrity is extremely encouraging for the development of a treatment for inflammatory conditions of the gut. The effects of fucoidan on gut inflammation may be in part related to the strengthening of tight junctions [101]. Tight junctions are essential in the lining cells of the gut to maintain integrity and prevent the “unauthorized” passage of larger molecules past the lumen of the gut. Once tight junction function is compromised, inflammation ensues [102], thus the strengthening of these connections is of great importance to gut health.

In summary, fucoidan continues to offer therapeutic activity as an anti-inflammatory agent, particularly in topical and gastrointestinal tract applications. Intravenous application of fucoidan fractions has yet to be developed as a regulated therapeutic. Enhanced oral uptake may offer a solution to the treatment of inflammatory conditions such as pancreatitis.

## 11. Fucoidan as a Treatment for Liver and Kidney Health

In the last review, we discussed the potential for fucoidan to limit diabetes-induced kidney damage and liver disease. Interestingly, this research clearly illustrates that orally delivered fucoidan has systemic effects, indicating that there is uptake and distribution of the fucoidan in the animal models.

Building on previous data showing the beneficial effects of fucoidan on kidney function, Wang *et al*., demonstrated that fucoidan from *Saccharina japonica* (*Laminaria japonica*) can limit kidney damage in a streptozoin-induced diabetes model [103]. Diabetes is often complicated by chronic, long-term kidney damage caused by high blood sugar. In this animal study, orally delivered fucoidan lowered blood sugar and decreased blood urea nitrogen levels. Renal output of nitrogen compounds was markedly preserved in the fucoidan-treated animals. This research illustrates a low toxicity dietary approach to treating or preventing diabetic kidney disease.

Fatty liver deposits are a major cause of silent disease in humans, whether caused by alcohol or not. Non-alcoholic fatty liver is the major cause of chronic liver disease [104] in western societies and has no known remedy. Kim *et al*., showed that mice exposed to a high-fat diet gained less weight when fucoidan was included in their diet [105]. Fatty liver deposits were also decreased, as were all blood lipids. Fucoidan derived from *Undaria pinnatifida* seaweed was included in the animals’ diet at a rate of only 1% or 2%—the equivalent of a supplement-sized dose for a human. This research places fucoidan as a candidate not only for weight control products, but for potentially improving cardiovascular and liver health. In related research, alcohol-induced fatty liver disease was investigated by Lim *et al.* [106]. Orally delivered *Fucus vesiculosus* fucoidan induced the protective hemoxygenase enzyme, in addition to reducing inflammatory markers in a mouse model. Similarly, alcohol induced fatty liver disease was attenuated by *Turbinaria decurrens* fucoidan in a rat model [107].

Fucoidan was also shown to benefit non-alcoholic liver disease caused by a pathogen. Hepatitis C is a viral infection that can lead to cirrhosis of the liver and hepatocellular carcinoma. Currently there is no available vaccine and the response rates to conventional treatments are less than ideal, however recent research suggests that there is significant potential for fucoidan in this area. A clinical study published in 2012 [108] showed that the oral administration of *Cladosiphon okamuranus* fucoidan was potentially usefully as a treatment. Fifteen patients with chronic hepatitis C, and HCV-related cirrhosis and hepatocellular carcinoma were treated with fucoidan (0.83 g/day) for 12 months. There were trends towards lower serum alanine aminotransferase levels (which correlated with HCV RNA levels). However, the improved laboratory tests did not translate into significant clinical improvement [108].

## 12. Fucoidan as a Modulator of Clotting

In the last review the activities of fucoidan in the clotting cascade were discussed. A recent excellent review article discusses the potential for this class of molecules as coagulation modifiers in detail [109]. Whilst fucoidans show considerable promise in this area, it may be difficult to replace existing products in the clinical market.

Fucoidan acts, in several respects, like the mammalian molecule heparin. Heparin is a widely used biological drug with antithrombotic activity, however the desired antithrombotic effect is very difficult to achieve without increasing the risk of haemorrhage, as heparin also has pronounced anticoagulant effects. A trial in mice showed that *Undaria pinnatifida* fucoidan (5 mg/kg iv) achieved an antithrombotic effect without any increases in clotting time, and could be developed for this purpose [110]. *Fucus vesiculosus* fucoidan had some prolongation of clotting time, once more demonstrating the differences between fucoidans.

Research into the concept of fucoidan as a pro-coagulant in hemophiliac blood missing clotting factors has also advanced, with new research on the pro- and anti-coagulant effects of fractions from *Fucus vesiculosus* [28]. This research showed that there is a minimum size of fucoidan polysaccharides required to exert an effect on coagulation. They found that fucoidan requires a minimal charge density of 0.5 sulfates per sugar unit and a size of 70 sugar units to demonstrate desired pro-coagulant activities for improvement of haemostasis in factor VIII/factor IX-deficient plasma.

A comparative study of fucoidans from *Laminaria japonica* with different molecular weight and sulfation patterns was carried out by Jin *et al.* [27]. In this case, the biological indicator was anticoagulant activity. They found that anticoagulant activity in the global clotting assay, activated partial thromboplastin time (aPTT), depended not only on the average molecular weight but also on the molar ratio of fucose to galactose. Molecular weight was found to have a more significant effect in the aPTT assay than the molar ratio.

Fucoidan from *Fucus vesiculosus* was shown to be a novel agonist for CLEC2 on platelets, causing the activation of platelet aggregation [111]. This reflects prior research by Durig [112] who also observed platelet activation *in vitro* by the same preparations and emphasizes the need to increase our understanding of fucoidan fractions before their use in clinical applications.

Lastly, as a result of its affinity for P-selectin, fucoidan has found a role as an imaging tool for atherosclerosis. In successive research studies, a group of researchers at INSERM in France used radiolabeled P-selectin to image thrombus *in vivo* [113,114]. This has particular utility in identifying the location of thrombi to allow targeted surgical intervention.

Thus, Undaria derived fucoidan has potential, particularly as an antithrombotic. If oral delivery can be achieved, this would be particularly valuable in the prevention of air travel induced deep vein thrombosis for example. The utility of specialized fucoidan fractions in imaging shows great potential for clinical application.

## 13. Biomaterial Development with Fucoidan

The functional properties of fucoidan make it an attractive target for the development of biomaterials. The main areas of interest seem to be drug delivery platforms, wound healing materials, bone building composites and *ex vivo* scaffolds for manipulation of stem cell growth, with a view to differentiation into particular tissue types.

The ability to control the release of active agents to the wound bed, and the potential to bind growth factors, are the key features of interest. Fucoidan-infused high water-content dressings were shown to improve wound healing in diabetics [115]. Details of two different types of fucoidan from *Fucus vesiculosus* and *Undaria pinnatifida* in multilayered films were examined and found to create different structural features, which may be of interest as potential drug delivery vehicles, lubricants and anti-fouling coatings [116].

Bone defects after removal of necrosed tissues require functional scaffolds for remineralisation. Fucoidan may offer a useful activity as it inhibits the differentiation of osteoclasts (bone resorbing cells). Fucoidan significantly inhibited NF-kappa B ligand (RANKL)-induced osteoclast formation and modulated the genes that control the resorption activity of osteoclasts [117]. Fucoidan has also been demonstrated to promote osteogenic differentiation of fat-derived stem cells [118,119] and of another mesenchymal cell type, amniotic fluid stem cells [118]. When fucoidan is incorporated into three dimensional structures posited for bone regeneration, promising early data has been obtained. One biomaterial composite of fucoidan and polycaprolactone (a biodegradable material commonly used in sutures) was investigated *in vitro*, and found to promote cellular mineralisation [120]. Addition of fucoidan to another type of macroporous structure composed of chitosan and alginate also promoted *in vitro* cellular mineralisation [121] as did fucoidan-modified hydroxyapatite composites examined by the same research group [122].

A French research group with an extensive track record of investigating fucoidan used *Fucus vesiculosus* fucoidan in a pullulan dextran-based macroporous scaffold to support human embryonic stem cell growth in heart cell structures, with the capacity to beat for a remarkable period of six months. This cardiac differentiating effect was mediated by the addition of growth factors which locally bound to and were then slowly released from the scaffold material [123]. It is possible that this type of scaffold could be applied as a therapeutic aid to recovery after heart muscle damage. A separate group of researchers also found that fucoidan supported the growth and differentiation of mesenchymal stem cells in a mouse hind limb ischemic model [124]. Interestingly, the fucoidan stimulated cells survived better in the model than the untreated cells. This indicates a potential “*ex vivo*” pre-treatment possibility in the emerging clinical field of mesenchymal stem cell treatments

In a similar line of research, Huang *et al*., have developed a method for the slow release of a normally short-lived chemotactic factor for all mesenchymal stem cells—stromal derived factor 1 (SDF1, CXCL12)—from chitosan-based nanoparticle materials containing fucoidan [125]. The mitogenic activity of these particles was confirmed *in vitro*, suggesting a potential application for attracting mesenchymal stem cells to particular tissue sites.

Overall, the use of fucoidan fractions in scaffolds, or as an *ex vivo* treatment, offers an exciting range of useful therapeutics that can be developed over the next decade.

## 14. Conclusions

Fucoidans continue to offer promise in a wide range of applications, some of which are covered in this review. Since the 2011 review on therapeutic applications for fucoidan, published original research has continued to increase. There is growing support for the role of fucoidan as an adjunct dietary therapy in cancer and inflammatory diseases. Fucoidan activity in promoting vaccine responses is a good example of currently available complementary therapeutic use. Appealing results on infectious diseases such as Leishmaniasis, as well inflammatory conditions deserve to be followed up with additional investigations, including clinical trials. It remains clear that each type of fucoidan needs to be screened and validated for a particular therapeutic activity. Assessment of pharmacokinetics, uptake and distribution are aspects that need consideration. This will become more practical thanks to the increased availability of detection and measurement techniques. Understanding the chemistry and structural variability of each type of fucoidan is a key part of developing successful therapeutic products.
